# Ambient air pollution and birth defects in Haikou city, Hainan province

**DOI:** 10.1186/s12887-014-0283-6

**Published:** 2014-11-22

**Authors:** Zhijiang Liang, Li Wu, Lichun Fan, Qingguo Zhao

**Affiliations:** Guangdong Women and Children Hospital, Guangzhou, China; Hainan Women and Children Hospital, Haikou, China

**Keywords:** Air pollution, Birth defects, PM_10_, SO_2_, NO_2_

## Abstract

**Background:**

Evidence shows exposure to ambient air pollution during pregnancy was associated with an increased risk of adverse birth outcomes, such as preterm birth, low birth weight and intrauterine growth retardation, but the results for birth defects have been inconsistent.

**Methods:**

The data on birth defects was collected from the Birth Defects Monitoring Network of Haikou city. Air pollution data for PM_10_, SO_2_ and NO_2_ were obtained from Haikou Environmental Monitoring Center. Logistic regression analysis was used to evaluate these associations.

**Results:**

The risk of birth defects was related to PM_10_ levels (adjusted OR = 1.039; 95% CI = 1.016-1.063) and SO_2_ levels (adjusted OR = 0.843; 95% CI = 0.733-0.969) for the second month of pregnancy. In the third month of pregnancy, the risk of birth defects was also related to PM10 levels (adjusted OR = 1.066; 95% CI = 1.043-1.090) and SO_2_ levels (adjusted OR = 0.740; 95% CI = 0.645-0.850).

**Conclusion:**

The study provides evidence that exposure to PM_10_ and SO_2_ during the second and third month of pregnancy may associated with the risk of birth defects.

## Background

Air pollution has become a common problem in many countries. Air pollution not only contributes to global warming but also has deleterious effects on the human health [1]. Children and pregnant women are especially vulnerable to the adverse impacts of air pollution [2]. Recent epidemiologic studies in different countries have indicated that there is association between ambient air pollution and adverse birth outcomes, such as preterm birth, low birth weight and intrauterine growth retardation [3-7]. However, studies in which the associations between ambient air pollution and birth defects are limited, and the periods of gestation when ambient air pollution may be associated with birth defects is also unclear. Smrcka et al. found that living in areas with industrial pollution was association with higher rates of congenital anomalies [8]. The study conducted in Southern California showed that ambient CO was positively associated with an increased risk of ventricular septal defects [9]. A recent study conducted in Brisbane found associations between O_3_ exposure and an increased risk of pulmonary artery and valve defects, and SO_2_ exposure and an increased risk of aortic artery and valve defects [10].

Birth defect is a part of a spectrum of adverse birth outcomes that may be associated with exposure to ambient air pollution. They have been a global public health issue, which are the main causes of early miscarriage, perinatal death and child disability. In China, the estimated prevalence is around 4% to 6% [11,12]. Approximately a quarter of perinatal deaths are affected by birth defects either directly or indirectly [13]. The etiology of congenital anomalies is unknown for as many as 60% cases, but about 6-8% is associated with exposure to environmental factors [14]. The study of birth defects is an important emerging field of environmental epidemiology.

There is growing evidence suggesting that ambient air pollution during pregnancy is associated with congenital anomalies. However, there has been limited research on the effect of air pollution during critical periods of pregnancy on birth defects. Based on air pollution monitoring network and birth defects surveillance system, we investigated whether maternal exposure to air pollution was associated with elevated birth defect risk in infants delivered between 2009 and 2011 in Haikou city, Hainan province. And we explore the sensitive gestations during which air pollution affect birth defects most significantly. We focused on air pollutants such as sulfur dioxide (SO_2_), nitrogen dioxide (NO_2_), and particles with an aerodynamic diameter of ≤10 um (PM_10_).

## Methods

### Subjects

The data on birth defects was collected from the Birth Defects Monitoring Network of Haikou city. The monitoring system is hospital-based registry, and the hospitals at the county level or above were selected to participate. The subjects monitored by the system included live births and stillbirths who were delivered in hospital after at least 28 weeks of gestation. The clinical diagnosis of birth defects was diagnosed within 7 days after delivery. Within this period, all diagnosed birth defects were required to be reported. We used unmatched case control study. Control infants were the other normal birth infants in this system. Control infants were selected from birth certificates, provided by the Haikou Department of Health Services. The data used in our study comprised all singleton births for the period of 1 January 2009 to 31 December 2011. During this period, there were 64100 singletons births included in our study. We received permission from Hainan Women and Children Hospital to use the data. The study was reviewed and approved by Guangdong Women and Children Hospital.

Information was collected from the birth certificates on gestation, birth weight, date of the last menstrual period (LMP), neonate gender, and age of mother.

### Exposure assessment

For the period January 2009 to December 2011, air pollution data for PM_10_, SO_2_ and NO_2_ were obtained from Haikou Environmental Monitoring Center. Hourly readings were obtained for PM_10_, SO_2_ and NO_2_. A daily average was calculated for PM_10_, SO_2_ and NO_2_.

We calculated the exposure parameters from the monthly average concentrations for the duration of pregnancies from 2009 through 2011. We also calculated the average concentration over the days of gestation for first, second and third month of gestation as this is the critical period of gestation associated with birth defects [15].

### Statistical methods

The effect of ambient air pollution on birth defects was estimated by logistic regression. We used odds ratio (OR) as a measure of the relation between exposure to air pollution and the risk of birth defects. We estimated adjusted OR using multiple logistic regression analysis and present the results as OR, along with 95% confidence interval (95% CI). We adjusted for risk factors that could potentially confound the relation between birth defects and air pollution. These factors were maternal age (<20, 20 ~ 24, 25 ~ 29, 30 ~ 34, ≥35 years), maternal race (Han, others), infant sex (male/female), birth weight (<2500/≥2500 g), gestational age (<37/≥37 weeks). We could not consider alcohol, tobacco and drug use during pregnancy, because these data are not recorded in the Haikou birth certificates. Statistical analysis was conducted using SPSS for Windows version 13.0.

## Results

### Characteristics of subjects

Characteristics of infants with or without birth defects are presented in Table 1. A large proportion of birth defects than control was male (*χ*^2^ = 3.70, *P* = 0.05), shorter gestational age (*χ*^2^ = 4118.32, *P* < 0.01), low birth weight (*χ*^2^ = 2521.36, *P* < 0.01) and Han race (*χ*^2^ = 15.22, *P* < 0.01).

**Table 1:** **Characteristics of subjects in Haikou city, Guangdong Province**

**Characteristics**	**Cases, n (%)**	**Controls, n (%)**	***χ*** ^**2**^	**P**
Infant sex				
Male	261 (51.28%)	35201 (55.53%)	3.70	0.05
Female	248 (48.72%)	28190 (44.47%)		
Maternal age (years)				
<20	20 (3.78%)	2419 (3.81%)	21.75	<0.01
20 ~ 24	109 (20.60%)	15786 (24.85%)		
25 ~ 29	195 (36.86%)	24197 (38.09%)		
30 ~ 34	110 (20.79%)	13707 (21.57%)		
≥35	95 (17.96%)	7424 (11.69%)		
Gestational age (weeks)				
<37	419 (79.21%)	4217 (6.63%)	4118.32	<0.01
≥37	110 (20.79%)	59354 (93.37%)		
Birth weight(g)				
<2500	327 (61.81%)	4070 (6.40%)	2521.36	<0.01
≥2500	202 (38.19%)	59501 (93.60%)		
Maternal race				
Han	516 (97.54%)	59307 93.29%)	15.22	<0.01
Other	13 (2.46%)	4264 (6.71%)		

### Air pollution

Descriptive statistics for air pollution levels during the study period are shown in Table 2. Levels of air pollutants had yearly variation. The levels of NO_2_ and PM_10_ showed no difference among three years (*P* > 0.05). The level of SO_2_ in 2011 was highest among three years, and there is difference among these years (*P* < 0.05).

**Table 2:** **Daily air pollution levels in Haikou (ppb)**

**Air pollutants**	**Year**	**N**	**Mean**	**SD**	**95% CI**		**F**	***P***
					**Lower**	**Upper**		
NO_2_	2009	365	15.88	4.75	15.39	16.37	0.638	0.529
	2010	365	15.43	6.14	14.80	16.06		
	2011	365	15.74	5.60	15.16	16.32		
PM_10_	2009	365	38.38	15.41	36.79	39.96	1.510	0.221
	2010	365	40.17	21.19	37.99	42.35		
	2011	365	40.62	18.35	38.73	42.51		
SO_2_	2009	365	7.03	3.91	6.63	7.43	9.753	<0.0001
	2010	365	6.72	3.58	6.35	7.09		
	2011	365	7.94	4.17	7.51	8.37		

SD = Std Deviation; CI = Confidence interval.

### Air pollution and the risk of birth defects

Table 3 shows the effect estimates from single-pollutant model. In the model, the risk of birth defects was related to PM_10_ levels, particularly in the third month of pregnancy (OR = 1.012; 95% CI = 1.003-1.021).

**Table 3:** **OR (95% CI) for birth defects during the first 3 months of pregnancy in single-pollutant model**

**Pollutants**	**β**	**SE**	***χ*** ^**2**^	***P***	**OR**	**OR 95% CI**	
						**Lower**	**Upper**
NO_2_							
1st month	−0.001	0.015	0.004	0.949	0.999	0.97	1.03
2nd month	−0.002	0.015	0.024	0.876	0.998	0.968	1.028
3rd month	−0.004	0.015	0.071	0.790	0.996	0.967	1.026
PM_10_							
1st month	0.008	0.004	3.113	0.078	1.008	0.999	1.017
2nd month	0.006	0.005	1.618	0.203	1.006	0.997	1.015
3rd month	0.012	0.005	6.825	0.009	1.012	1.003	1.021
SO_2_							
1st month	0.027	0.024	1.315	0.252	1.028	0.981	1.077
2nd month	0.025	0.024	1.063	0.303	1.025	0.978	1.075
3rd month	0.03	0.025	1.484	0.223	1.030	0.982	1.081

*P* < 0.05 indicates the difference was statistically significant.

Table 4 shows the effect estimates from three-pollutant model. In the three-pollutant models, the risk of birth defects was related to PM_10_ in the third month of gestation, after adjusting for other two air pollutants.

**Table 4:** **OR (95% CI) for birth defects during the first 3 months of pregnancy in three-pollutant models**

**Pollutants**	**β**	**SE**	***χ*** ^**2**^	***P***	**OR**	**OR 95% CI**	
						**Lower**	**Upper**
1st month							
NO_2_	−0.017	0.021	0.641	0.424	0.984	0.945	1.024
PM_10_	0.013	0.009	1.953	0.162	1.013	0.995	1.031
SO_2_	−0.015	0.056	0.075	0.784	0.985	0.883	1.098
2nd month							
NO_2_	−0.019	0.02	0.909	0.340	0.981	0.943	1.021
PM_10_	0.006	0.009	0.401	0.527	1.006	0.988	1.024
SO_2_	0.018	0.054	0.116	0.734	1.019	0.917	1.132
3rd month							
NO_2_	−0.025	0.02	1.492	0.222	0.976	0.937	1.015
PM_10_	0.024	0.009	8.039	0.005	1.024	1.007	1.042
SO_2_	−0.051	0.051	1.02	0.313	0.95	0.86	1.049

*P* < 0.05 indicates the difference was statistically significant.

Table 5 summarizes the results of logistic regression analysis from each single pollutant model, adjusting for maternal age, maternal race, infant gender and birth weight. In the single-pollutant model, the adjusted OR for PM_10_ was 1.021 (95% CI = 1.008-1.034) for the third month of pregnancy.

**Table 5:** **Adjusted OR (95% CI) for birth defects during the first 3 months of pregnancy in single-pollutant model**

**Pollutants**	**β**	**SE**	***χ*** ^**2**^	***P***	**OR**	**OR 95% CI**	
						**Lower**	**Upper**
NO_2_							
1st month	0.015	0.021	0.529	0.467	1.015	0.975	1.058
2nd month	−0.003	0.021	0.024	0.876	0.997	0.956	1.039
3rd month	−0.001	0.022	0.004	0.949	0.999	0.957	1.042
PM_10_							
1st month	0.005	0.006	0.54	0.463	1.005	0.993	1.017
2nd month	0.012	0.006	3.85	0.050	1.012	1.000	1.025
3rd month	0.021	0.006	10.368	0.001	1.021	1.008	1.034
SO_2_							
1st month	−0.003	0.034	0.006	0.938	0.997	0.934	1.066
2nd month	0.005	0.035	0.018	0.895	1.005	0.938	1.076
3rd month	−0.005	0.036	0.022	0.882	0.995	0.928	1.066

*P* < 0.05 indicates the difference was statistically significant.

Table 6 summarizes the results of logistic regression analysis from three-pollutant models, adjusting for maternal age, maternal race, infant gender and birth weight. In the three-pollutant models, the risk of birth defects was related to PM_10_ levels (adjusted OR = 1.039; 95% CI = 1.016-1.063) and SO_2_ levels (adjusted OR = 0.843; 95% CI = 0.733-0.969) for the second month of pregnancy. In the third month of pregnancy, the risk of birth defects was also related to PM_10_ levels (adjusted OR = 1.066; 95% CI = 1.043-1.090) and SO_2_ levels (adjusted OR = 0.740; 95% CI = 0.645-0.850).

**Table 6:** **Adjusted OR (95% CI) for birth defects during the first 3 months of pregnancy in three-pollutant models**

**Pollutants**	**β**	**SE**	***χ*** ^**2**^	***P***	**OR**	**OR 95% CI**	
						**Lower**	**Upper**
1st month							
NO_2_	0.033	0.027	1.446	0.229	1.033	0.98	1.09
PM_10_	0.020	0.012	2.807	0.094	1.020	0.997	1.045
SO_2_	−0.130	0.074	3.115	0.078	0.878	0.76	1.015
2nd month							
NO_2_	0.001	0.027	0.002	0.964	1.001	0.949	1.056
PM_10_	0.038	0.012	10.995	0.001	1.039	1.016	1.063
SO_2_	−0.171	0.071	5.786	0.016	0.843	0.733	0.969
3rd month							
NO_2_	0.008	0.028	0.088	0.767	1.008	0.954	1.066
PM_10_	0.064	0.011	32.092	<0.001	1.066	1.043	1.090
SO_2_	−0.301	0.07	18.386	<0.001	0.740	0.645	0.850

*P* < 0.05 indicates the difference was statistically significant.

## Discussion

Birth defects are the main causes of perinatal death, missed abortion, and child disabilities and have been a global public health issue. They are generally caused by several factors. Risk factors which contribute to birth defects include genetic factors, environmental factors, chemicals and maternal elements [16]. Our study investigated the possible association between ambient air pollution and risk of birth defects. The study contributes to a growing body of epidemiologic literatures on the adverse reproductive effects of air pollution exposure. We found mixed results across all analyses. In the second month of pregnancy, the risk of birth defects was related to PM_10_ levels (adjusted OR = 1.039; 95% CI = 1.016-1.063) and SO_2_ levels (adjusted OR = 0.843; 95% CI = 0.733-0.969). In the third month of pregnancy, the risk of birth defects was also related to PM10 levels (adjusted OR = 1.066; 95% CI = 1.043-1.090) and SO_2_ levels (adjusted OR = 0.740; 95% CI = 0.645-0.850). The most susceptible time periods in pregnancy for the effects of SO_2_ and PM_10_ were the second and third month of gestation. SO_2_ is a major air pollutant produced from coal and oil combustion, which has significant impacts upon human health. Particulate matter includes a variety of pollutants that are suspended as particles in the air, such as road dust, ash and smoke.

There have been inconsistent results across previous studies that have examined associations between ambient air pollution and birth defects. And, each of these studies found only one or two associations. For example, in Brisbane, Australia, exposure to PM_10_, NO_2_, SO_2_, CO and O_3_ was examined and results showed that O_3_ was associated with an increased risk of aortic artery and valve defects and SO_2_ was associated with cleft lip with or without cleft palate [10]. The study conducted in Atlanta, Georgia, examined 12 types of cardiovascular malformations and five pollutants (CO, NO_2_, PM_10_, SO_2_, and O_3_) and found a statistically significant association between PM_10_ and patent ductus arteriosus [17]. The results from the Texas study revealed that the positive associations between CO and Tetralogy of Fallot, PM_10_ and atrial septal defect, and SO_2_ and ventricular septal defect [18]. In South California, exposure to ambient PM_10_, NO_2_, O_3_ and CO during each of the first three months of pregnancy was examined and the results only showed the association between CO and increased risk of cardiac ventricular septal defects, O_3_ and an increased risk of aortic artery and valve defects [9].

Compared with previous studies, one potentially important difference is that we estimated the effect of air pollution as categorical exposure, whereas other studies estimated the effect of air pollution as continuous exposure [17,19]. Using a categorical exposure puts no restrictions on the shape of the exposure-risk relationship, but reduces statistical power.

Previous studies have shown that maternal exposure to air pollutants can have teratogenic effects. Possible mechanisms of air pollutants on birth defect remain speculative. Air pollutants might be involved in the development of skeletal malformation via hemodynamic, anoxic events, oxidative stress, and toxicity to certain cell populations during pregnancy [9].

In our study, we observed an increased risk of birth defects for second-month and third-month PM_10_ exposures. Thus, the timing of PM_10_ exposure is consistent with embryo development. However, we also found a reduced risk associated with increased SO_2_ exposures in the second and third month, which might suggest these fetuses are vulnerable when exposed to SO_2_. We can not rule out ascertainment bias due to prenatal diagnosis as well as selective abortion of fetuses with birth defects. This observation on PM_10_ and SO_2_ might suggest that a different effect of air pollutants on birth defects.

Our study had several strengths. We conducted a large, population-based analysis using a high-quality birth defects monitoring network with air pollution monitoring data from Haikou Environmental Monitoring Center. To allow adjustment for the possible effect of weather on birth defects, some meteorologic factors, such as daily average temperature, humidity data were collected from Haikou Meteorological Bureau. We adjusted for several confounders in the logistic regression analysis to eliminate the factors as a potential explanation for our results. Our study used a large number of birth records based on birth certificates, which reduces uncertainties due to selection bias which is more common in smaller studies. Despite these efforts, however, residual confounding is possible. First, the monitoring period was 28 weeks’ gestation to 6 days after delivery, and babies with birth defect detected more than 7 days after delivery would be missed. And we can not study the effect of air pollution on infants with birth defect more than 7 days after delivery. Second, we assigned concentrations of air pollutants from the monitoring sites to all women residing in a large area rather than measuring each pregnant woman’s exposure to each pollutant during pregnancy. Second, several factors including maternal smoking, occupational exposures, and vitamin supplement use might be potential risk factors for birth defect. We were unable to evaluate, because they are not adequately reported on Haikou birth certificates.

## Conclusion

Our study contributes to a growing body of epidemiologic literature on the adverse reproductive effects of air pollution exposure. Our results suggest that exposure to PM_10_ during the second and third month of pregnancy may contribute to the occurrence of birth defects. To date, a limited body of evidence has linked maternal exposure to ambient SO_2_, NO_2_ and PM_10_ to the occurrence of birth defect. Further studies are needed to address these associations.
